# Transcatheter closure in preterm infants with patent ductus arteriosus: feasibility, results, hemodynamic monitoring and future prospectives

**DOI:** 10.1186/s13052-023-01552-2

**Published:** 2023-11-06

**Authors:** Gaia Francescato, Daniela Doni, Giuseppe Annoni, Irma Capolupo, Elena Ciarmoli, Iuri Corsini, Italo Francesco Gatelli, Sabrina Salvadori, Alberto Testa, Gianfranco Butera

**Affiliations:** 1 Neonatal Intensive Care Unit, Fondazione IRCCS Ca’ Granda Ospedale Maggiore Policlinico, Milan, Italy; 2 Neonatal Intensive Care Unit Fondazione IRCCS San Gerardo Dei Tintori, Monza, Italy; 3 Pediatric Cardiology, Regina Margherita Children’s Hospital, Turin, Italy; 4 Neonatal Intensive Care Unit, Ospedale Pediatrico Bambino Gesù, Rome, Italy; 5 U.O. Di Neonatologia, Patologia Neonatale E Pediatria, ASST Della Brianza, P.O. Vimercate, Vimercate, Italy; 6 Division of Neonatalogy, Careggi University Hospital of Florence, Florence, Italy; 7 Division of Neonatology and Neonatal Intensive Care Unit, ASST Grande Ospedale Metropolitano Niguarda, Milan, Italy; 8 Women and Child Health Department, Neonatal Intensive Care Unit, Azienda Ospedaliera -Università Di Padova, Padua, Italy; 9 Sapienza School for Advanced Studies, Sapienza University of Rome, Rome, Italy; 10 Cardiology, Cardiac Surgery and Heart Lung Transplantation; ERN GUARD HEART: Bambino Gesù Hospital and Research Institute, IRCCS, Rome, Italy

**Keywords:** Preterm infants, Patent ductus arteriosus, Transcatheter closure

## Abstract

Ductal patency of preterm infants is potentially associated with long term morbidities related to either pulmonary overflow or systemic steal. When an interventional closure is needed, it can be achieved with either surgical ligation or a catheter-based approach.

Transcatheter PDA closure is among the safest of interventional cardiac procedures and it is the first choice for ductal closure in adults, children, and infants weighing more than 6 kg. In preterm and very low birth weight infants, it is increasingly becoming a valid and safe alternative to ligation, especially for the high success rate and the minor invasiveness and side effects. Nevertheless, being it performed at increasingly lower weights and gestational ages, hemodynamic complications are possible events to be foreseen.

Procedural steps, timing, results, possible complications and available monitoring systems, as well as future outlooks are here discussed.

**Supplementary Information:**

The online version contains supplementary material available at 10.1186/s13052-023-01552-2.

## Background

The ductus arteriosus in preterm infants remains patent for ten or more days after birth in more than 50% of all infants born before 30 weeks’ gestation [1, 2]. Ductal patency is potentially associated with long term morbidities related to either pulmonary overflow or systemic steal. Nevertheless, no causal relationship has been proven between patent ductus arteriosus (PDA) and increased mortality or specific morbidities, except for retrospective studies [3, 4]. After more than 40 years of clinical research, including many randomized controlled trials (RCTs) many questions remain unanswered. Strategies for its management which include medical pharmacological approach, interventional approach and conservative approach, remain a subject of great controversy because of the paucity of evidence that interventions reduce adverse outcomes [5–7].

### Medical and management approaches

In a meta-analysis of 58 RCTs inclusive of 6028 subjects, medical prophylaxis or treatment of the PDA was not associated with any significant reduction in neonatal mortality or in measured morbidities [8]. Nevertheless, since RCTs included infants in a wide range of GAs, had widely varying PDA definitions including PDA diameter alone, and provided open-label treatment, it is difficult to draw inferences on clinical outcomes based on the results of these trials.

Medical therapy aimed at hemodynamically significant PDA (HsPDA) closure is based on the administration of either non-steroidal anti-inflammatory drugs (NSAIDs) like indomethacin or ibuprofen, or paracetamol. However, which dose or which drug is recommended for each infant is far to be established.

The modern conservative approach has gained interest since the early 2000s. It is driven by concerns over unnecessary and potentially harmful interventions, without demonstrated benefits other than ductal closure itself [7]. This approach includes a variety of actions, including positive pressure for respiratory support, mild fluid restriction [9], selective diuretic use, avoiding anemia and providing adequate nutrition until the duct is no longer hemodynamically significant.

### Non-medical approaches

Even if not considered as first line option, when medical treatments fail and the patient is still suffering from the hemodynamic impact of a large PDA, then an interventional closure is considered. This may be obtained by a surgical or a catheter-based approach.

Surgical ligation of the PDA historically has been the first alternative to failed pharmacological treatment and it is usually performed through left thoracotomy. However, it is at increased risk of mortality and significant morbidities in this vulnerable group of infants. Thirty days mortality rate has been reported around 5–8%. Surgical ligation has been reported to be associated to bleeding, infection, vocal paresis, hence to gastroesophageal reflux disease (GERD) and need for prolonged intubation and mechanical ventilation [10]. The post-operative course of preterm infants undergoing surgical ligation of PDA is often complicated by post ligation cardiac syndrome (PLCS) with decreased cardiac output and hemodynamic instability in 28%–45% of infants despite targeted milrinone prophylaxis. It has also been associated with an increased incidence of bronchopulmonary dysplasia (BPD), retinopathy of prematurity (ROP) and neurodevelopmental impairment in comparison with delayed ligation in a selected population. Nevertheless, controversies remain whether these are related to surgical ligation or prolonged exposure of preterm infants to PDA itself or possible associated co-morbidities [11, 12].

Transcatheter PDA closure is among the safest of interventional cardiac procedures and is the first choice for ductal closure in adults, children, and infants ≥ 6 kg. A device is deployed by a transcatheter approach to seal the opening between the aorta and the pulmonary artery, thereby restoring normal blood flow.

In a recent study, Wilson et al. evaluated success and complication rates of transcatheter closure of PDA in 141 adult patients. They reported a 100% success rate and no major complications. Six percent of treated patients had a small residual shunt, and only 2 patients had a residual leak on echocardiography at follow-up. The authors concluded that transcatheter PDA closure is very effective in adults across all duct morphologies and associated with a very low complication rate [13].

In another study, Sudhakar et al. provided comprehensive data on the safety and efficacy of transcatheter closure of PDA in an adult and adolescent population, thus confirming the feasibility of this technique in a younger population. Of 70 PDA device closure cases, 64 were carried out using occluders (ADO-I and II, Lifetech, Cardi-O-Fix). Device success was achieved in all including patients with very large PDAs, and no major complications occurred. At follow up, complete closure was observed in all patients [14].

Therefore, this success in adults and adolescents paved the way for transcatheter closure in preterm infants.

### Transcatheter PDA occlusion in preterm infants

In recent years, the use of transcatheter PDA closure has gained wide attention as a less invasive alternative to surgical ligation and a more effective treatment option than medical therapy for extremely low birth weight (ELBW) infants [15].

In two studies, Zahn et al. demonstrated that transcatheter PDA closure can be successfully performed in preterm neonates using currently available technology with a high success rate and a low incidence of complications. In addition, the author introduced a new transvenous method that utilizes both echocardiography and careful employment of fluoroscopy to circumvent arterial access in this vulnerable group of patients [16, 17].

**Fig. 1:**
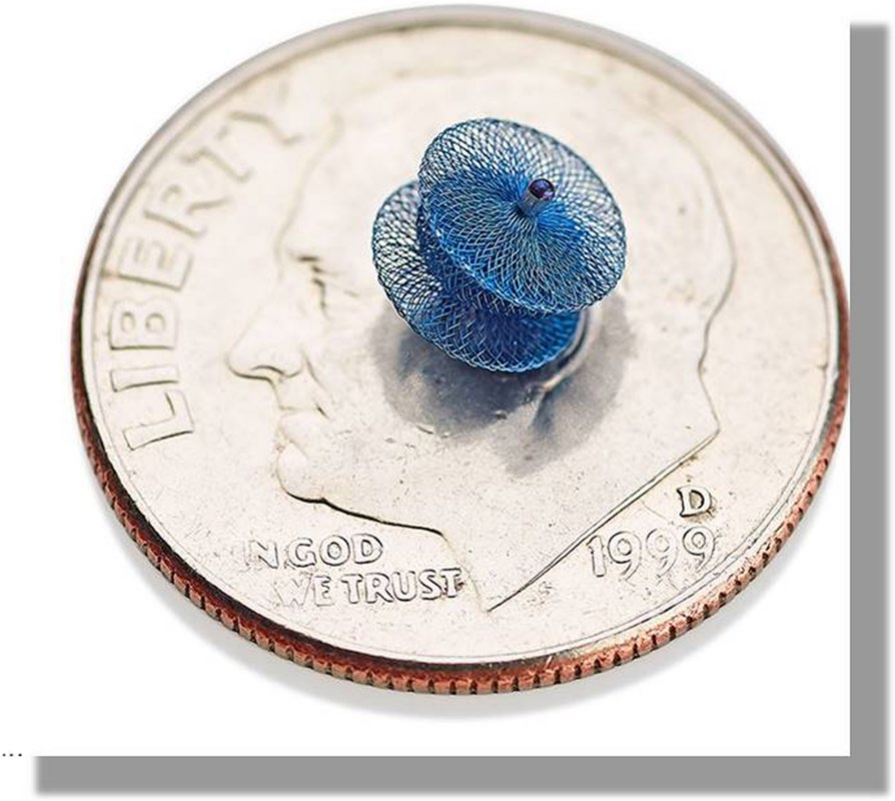
Amplatzer Piccolo™ Occluder (APO, Abbott Structural Heart, Plymouth, MN, USA)

Since 2019, FDA and EU approved the device Amplatzer Piccolo™ Occluder (APO, Abbott Structural Heart, Plymouth, MN, USA) for the treatment of preterm patients [18, 46]. It has a particular design for fetal ductus morphology, elongated-tubular PDA with a narrowing on the pulmonary side, (Hockey stick morphology) [19, 20] (Fig. 1). In the United States, a single arm, prospective, multicenter, non-randomized study was conducted to assess its efficacy in patients weighing 700 g or more. It resulted in an implant success rate of 95.5% overall and 99% in patients weighing less than or equal to 2 kg [18].

In order to proceed with transcatheter closure the duct must be longer than 3–5 mm with a maximal diameter of 4 mm [21].

### Transcatheter procedure

Cath lab settings is extremely important for treating preterm infants; in fact, this procedure needs a multidisciplinary team that include neonatologists, anesthesiologists, pediatric cardiologists, and specialized nurses of catheterization laboratory and neonatal intensive care unit.

Before the procedure is performed, a checklist is shared with neonatologists and anesthesiologists to reduce potential risks: recent blood exams are verified, one red blood cells bag should be available, and the ventilator in catheterization laboratory should be specific for preterm infants. Temperature control of the preterm infant is mandatory as well as the availability of a neonatal ultrasound probe. If possible, the temperature of catheterization laboratory should be raised up to avoid cooling of the preterm infant.

When the team, and especially the interventional cardiologist is more than confident with the procedure, the transcatheter closure can be done in neonatal intensive care unit with portable fluoroscopic unit.

Before starting the procedure, an accurate Echocardiography is performed to confirm the anatomy of the PDA and measurements [22, 23].

To reduce potential complications, the procedure should be concluded in 60–90 min. Therefore, in normal settings, right heart catheterization with measurement of pressure, PVR and CI is avoided. When transcatheter closure is completed, babies must return in intensive care unit as soon as possible. A surgical back-up should be available.

The procedure is performed under general anaesthesia, and 4-French femoral vein access is required. Arterial access is contraindicated for high risk of vascular complications [24]. Vascular accesses are echo guided inserted.

The first critical step is crossing the tricuspid valve with guidewire and catheter. In order to do so, a 3.3 Fr right coronary catheter (JR Mongoose) is advanced up to the annulus of the tricuspid valve. A 0.0014″ J tip coronary guidewire is then advanced trough the tricuspid valve into the right ventricle, main pulmonary artery, ductus arteriosus and descending aorta.

Further on, over the coronary guidewire a telescopic system is advanced. It includes the LP torqVue delivery system and a microcatheter. By using this system, the risk of entrapping the tricuspid valve is extremely low.

**Fig. 2:**
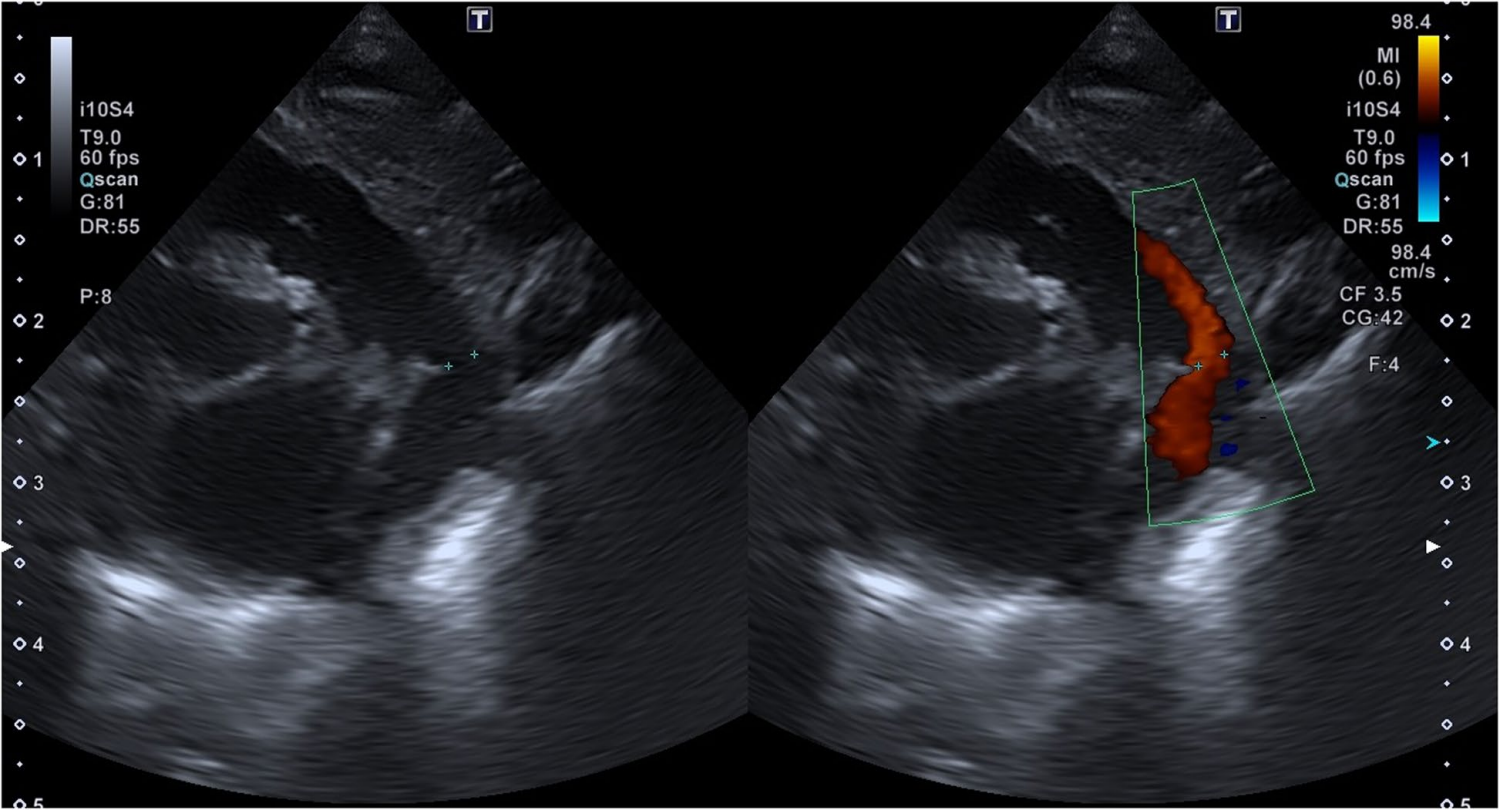
Echocardiographic measurement of the patent duct ‘s diameter in a short axis scan

**Fig. 3:**
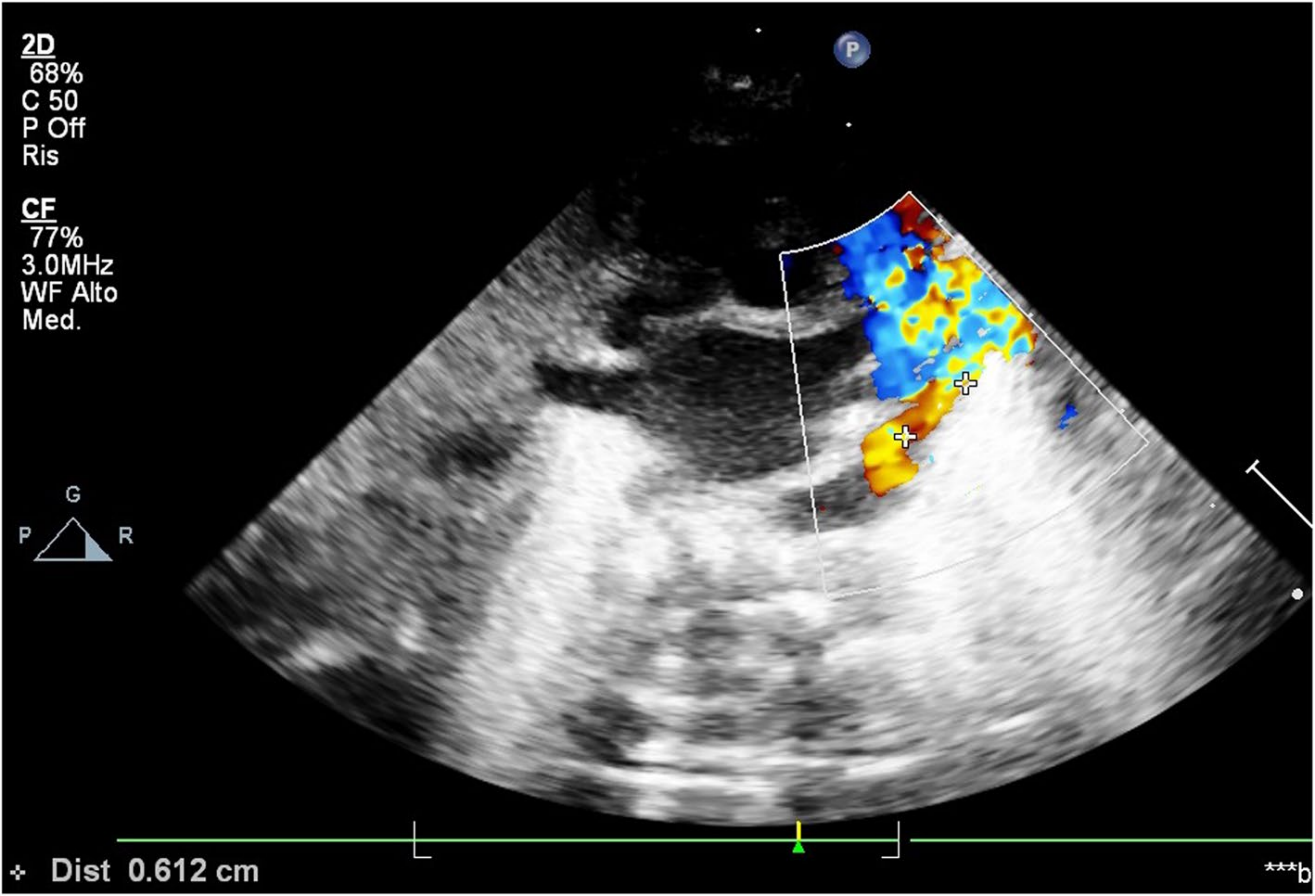
Echocardiographic measurement of the length of the patent duct in a low short axis scan

**Fig. 4:**
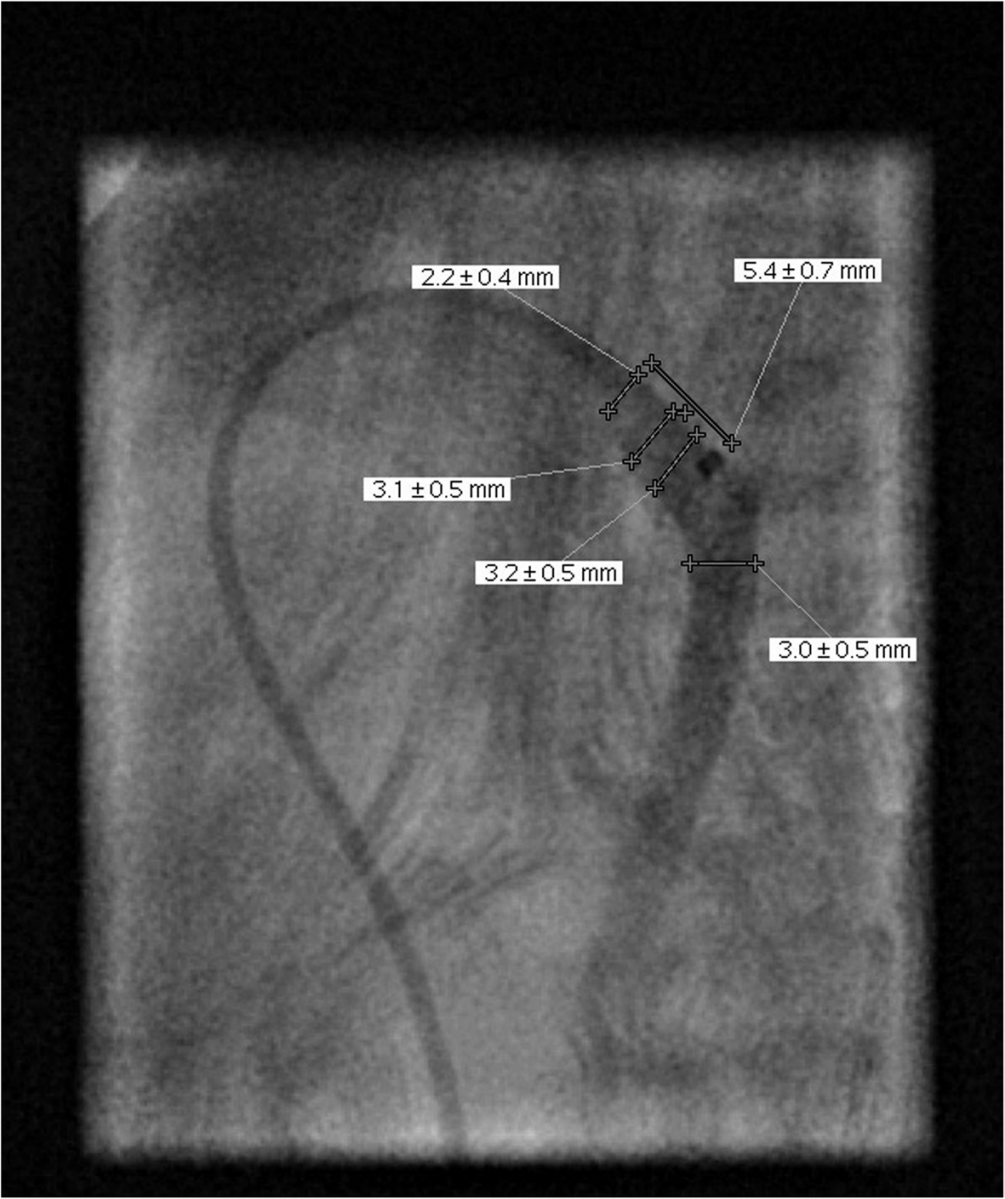
Angiographic latero-lateral view and measurement of the patent duct after injection of contrast

Single hand injection of contrast is performed across the PDA; 1 ml/kg is enough for complete view of PDA in latero-lateral projection. Echocardiographic and angiographic measurements are obtained to finally choose the device (Figs. 2, 3, 4). The device is chosen by using two parameters: PDA measurements and weight of the preterm.

In preterm infants weighing less than 2 kg, the Piccolo Occluder must be placed completely inside the ductus. Conversely, in infants weighing more than 2 kg the device can be placed with external disks in aorta and in pulmonary artery [18, 19, 22]. Amplatzer Piccolo Occluder ™ (APO) is advanced and deployed in correct position across the PDA with echocardiography monitoring [25, 23] and fluoroscopy control (LL projection) (clip 1, clip 2). Trachea and oesophagus are good markers for correct positioning of the device. A proper device orientation at fluoroscopy shows coaxially aligned with the long axis of the ductus and pointing toward 10 o’clock on a 90° in lateral fluoroscopy view [18, 19].

**Fig. 5:**
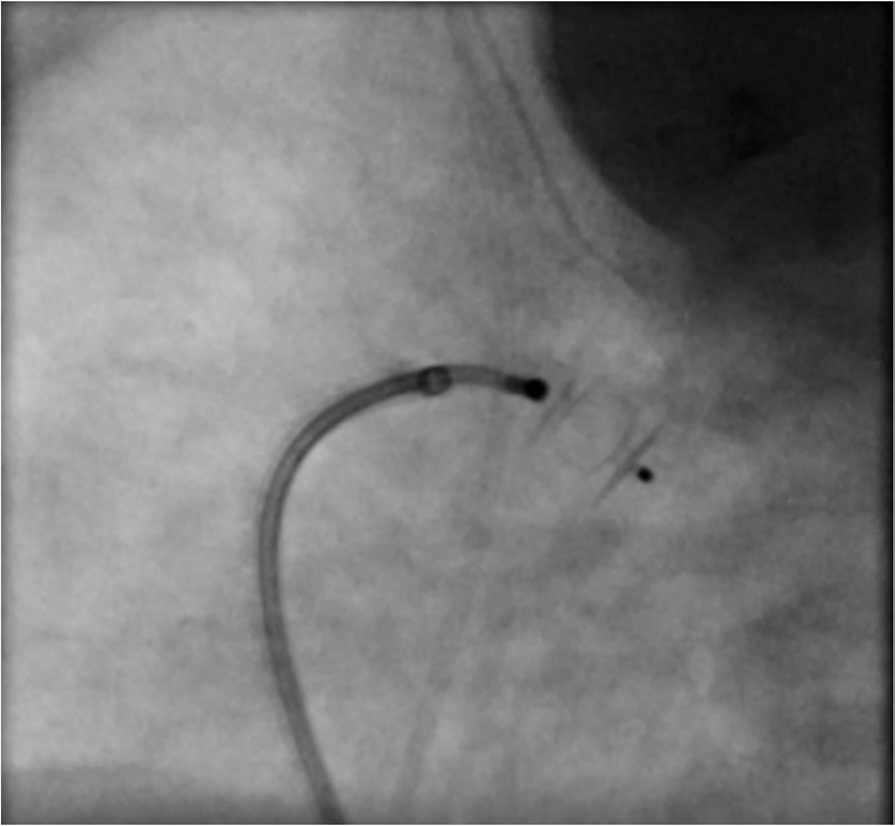
Echocardiographic evaluation right after the release of the device

Residual shunt, aortic coarctation, protrusion in left pulmonary artery are excluded with echocardiography. The device is than released when in correct position (Clip 3, Fig. 5).

### Results of the transcatheter procedure

Transcatheter closure of the PDA in preterm infants is therefore a feasible and safe technique with reported success rate of 98% [26, 27] with a very low rate of major adverse events as reported in meta-analysis studies [16, 17, 25, 28].

Sathanandam et al. summarized the current consensus guidelines for the prevention and management of periprocedural complications of transcatheter PDA closure with the Amplatzer Piccolo Occluder in ELBW infants [19]. Despite the low frequency of periprocedural complications, severe reported complication are dissection of inferior vena cava, cardiac perforation (rare ≃ 0.8%), these risks are minimized using a 0.014 guide wire [29]. Less severe and more frequent complications are protrusion of the proximal disk at the pulmonary end causing left pulmonary artery (LPA) stenosis (1.2%), protrusion of the distal disk in aorta causing aortic coarctation (1.2%), device embolization (more frequent in patients with large PDA) (2.8%), tricuspid regurgitation (mild-trivial ≃ 2%). There is also a risk of residual shunt or recurrence of PDA, which may require additional interventions. Mortality is 0.6% [30–33].

### Surgical versus transcatheter PDA closure in preterm infants

A recent metanalysis [34] that screened 97 studies, 8 of which met the eligibility criteria, with a total of 756 preterm infants below 2000 g birthweight, aimed at assessing the safety and efficacy of transcatheter closure (TC) when compared to surgical ligation (SL) in preterm infants with PDA. Compared to TC, SL had higher mortality rates. No difference was seen in post-procedural complication rate, mean duration of post-procedural mechanical ventilation, hospital stay length or neonatal intensive care unit stay length.

As to renal function, a single center retrospective study observed a significant improvement in renal function after transcatheter closure, even with the use of contrast, comparable to those of patients who underwent surgical closure [35]. Table 1 summarizes pros and cons of surgical and transcatheter procedures.

**Table 1:** Pros and cons of surgical and transcatheter procedures

Categories	Transcatheter PDA Closure	Surgical PDA Closure
Performing operator	Interventional cardiologists	Pediatric cardiac surgeons
Expertise and technique	Pros: - Minimally invasive procedure - Completely intravascular procedure	*Pros*: - Technique with a longer history and follow-up - Can be performed at bedside
	*Cons*: - Limited availability of experienced operators - Need to transfer the patient in a dedicated catheterization laboratory	*Cons*: - Requires specialized training in pediatric cardiac surgery - Involves a larger incision
Safety	*Pros*: - Lower risk of infection and bleeding - Lower risk of Post ligation syndrome - Faster Pulmonary score recovery - Shorter recovery time and hospital stay - No trauma on tissues and organs surrounding the duct	*Pros*: - Immediate confirmation of closure
	*Cons*: - Risk of vascular injury, tricuspid injury and device embolization - Limited data on long-term outcomes in premature infants	*Cons*: - Higher risk of infection and bleeding - Long recovery time and hospital stay - Potential for scar formation and chest wall deformity - Potential vocal cord paralysis
Future outlook	Bedside procedure under echocardiographic and fluoroscopic monitoring	

### When is the right time to implant a transcatheter occluder?

There is still debate on how to evaluate a hemodynamically significant duct. Consistent PDA scores [36] should be developed in order to ensure that infants at greatest risk for adverse ductal consequences are included.

Ideal timing of transcatheter closure is yet to be determined. As previously stated, both transcatheter and surgical procedures are mostly (but not only) performed after medical treatment failure.

Besides anecdotal findings and single center experiences which could suggest that the time lapse a preterm infant is exposed to the effects of a significant ductal shunting could be directly related to the risk of developing morbidities such as BPD [3] and acute renal failure [37], no clear evidence can support a specific recommendation in terms of timing.

Nevertheless, Regan et al.’s subgroup analysis of their cohort [27] demonstrated a shorter hospitalization in babies younger than 4 weeks of life at the time of transcatheter closure.

It is important to note that not all cases of PDA can be treated with transcatheter closure, and it is crucial to consider individual patient factors when determining the appropriate treatment strategy. Therefore, a multidisciplinary team consisting of neonatologists, cardiologists, and pediatric cardiac surgeons is necessary to make informed decisions about treating preterm infant with PDA. Surgical closure remains a viable option for infants with complex anatomy or significant comorbidities.

### Hemodynamic monitoring of patients with PDA pre, during and after the procedure

Infant hemodynamic balance depends on cardiac output (CO) and systemic vascular resistances (SVR). McNamara et al [38] described a population of preterm infants weighting between 995 and 1318 g who closed the PDA with percutaneous device. They showed that one hour after PDA closure there was a significant decrease in stroke volume (SV), consequent to a reduced left ventricular pulmonary venous return and an increase of arterial elastance, due to a loss of low resistance pulmonary vascular bed circuit, with maintained diastolic blood pressure (BP). After PDA closure, the significant increase of arterial elastance would be expected to generate significant increase of BP, but the pronounced drop in preload determines a low cardiac output and, consequently, an apparently stable diastolic pressure, so that the clinicians may fail to recognize significant changes in left ventricular function.

For this reason, monitoring CO and SVR in preterm infants undergoing percutaneous PDA closure is very important and multiple tools to identify short-term myocardial dysfunction are needed to set an early treatment.

The use of targeted neonatal echocardiography is useful to early detection infants at risk of PLCS: CO < 200 ml/kg/min within 1 h of PDA ligation may predict subsequent cardiorespiratory compromise and the need of inotropic agents, and administration of i.v. milrinone is associated with improved postoperative stability [39]. In the same way, post percutaneous PDA closure, early functional echocardiography allows to detect the cases of inability of the myocardium to adapt to sudden changes in loading condition.

Post PDA closure, interstitial pulmonary oedema, sustained to exposure to high volume left-to-right shunt, is reduced and it has been demonstrated a reduction in lung ultrasound score (LUS) 1 h after surgical intervention [40]. Moreover, the drop of LUS is correlated to lowering in CO, suggesting that the lung ultrasound may be a useful tool to guide monitoring of the pulmonary disease and the cardiac function also after PDA device position.

Electrical cardiometry (EC) is a non-invasive method that measures thoracic electrical bioimpedance and derives hemodynamic parameters such CO, SVR and contractility index; application during PDA ligation has demonstrated that abrupt diversion of a ductal shunting contributes to hemodynamic aberrations in VLBW infants and that increased SVR, decreased preload and impaired left ventricular performance might be the principal causes of it [41]. In preterm infants undergoing percutaneous PDA closure, EC is useful to record hemodynamic changes, to recognize the acute increase of SVR and their trend: in a recent paper, it seems that long persistence of high SVR could be correlated to circulation impairment and drop of CO, resulting in development of PLCS, while a rapid normalization to preoperatory value of SVR may be a good indicator of cardiorespiratory stability [42]. Near-infrared spectroscopy (NIRS) measuring the difference in the absorption spectra of oxygenated and deoxygenated hemoglobin to indirectly assess flow is a valid continuous assessment of regional tissue oxygenation (rSO2) and it has become available and gained evidence-based application in neonatal intensive care [43].

In pediatric and neonatal cardiac surgery, it can be applied perioperatively to monitor regional cerebral tissue oxygenation and perfusion.

Cerebral and renal oxygen saturation and extraction do not seem to be affected by an HsPDA or by retrograde diastolic blood flow in the descending aorta [44].

After PDA ligation and transcatheter closure in preterm infants, an initial short-term decrease followed by an increase in cerebral rStO2 can been observed [45], due to the perturbation of cerebral blood flow; future research is needed to understand the effects on cerebral oxygenation during transcatheter closure of ductus arteriosus.

Little is known about hemodynamic complications of transcatheter closure. The lower incidence of hemodynamic imbalance, need for inotropes and ventilatory support might be due to demographic parameters. Infants that undergo surgical ligation are generally smaller and younger in gestational age (GA) and in days of life (DOL).

### Future outlook

Many steps have been done in the recent years in term of a less invasive procedure with a higher rate of success. However, there are still several critical steps related to the transfer of the patients in the catheterization laboratory. There are some efforts in performing the procedure at the bedside in the neonatal intensive care unit under echocardiographic and fluoroscopic monitoring. However, several units in the world are trying to do the next step that is a totally echocardiographic guided procedure at the bedside in the neonatal intensive care unit [46]. This will reduce the logistic burden and the impact of a transfer on the preterm infants’wellbeing.

## Conclusions

In conclusion, PDA transcatheter closure is increasingly becoming a valid and safe alternative to ligation, especially for the minor invasiveness and side effects. Nevertheless, with the reduction of the weight and gestational age of the newborns to which it is performed, hemodynamic complications are possible events to be foreseen.

Despite the usefulness of this method in managing preterm neonates, there are still limitations to the procedure, and surgical closure may still be a viable option depending on the individual case. It is therefore important to continue researching and enhancing the device and delivery system to maximize its potential benefits for this vulnerable population.

Furthermore, hemodynamic monitoring should include the integration of multiple systems (functional echocardiography, lung ultrasound scan, EC, NIRS) to recognize soon those infants with ventricular dysfunction, who may benefit from early treatment.

### Supplementary Information

**Additional file 1:** **Clip 1.** Echocardiographic evaluation of the device’s advancement and deployment in a correct position across the PDA.

**Additional file 2:** **Clip 2.** Fluoroscopic evaluation of the device’s correct positioning for release.

**Additional file 3:** **Clip 3.** Echocardiographic evaluation right after the release of the device.

## Data Availability

N/A.

## Abbreviations

APO: Amplatzer piccolo occluder
BP: Blood pressure
BPD: Bronchopulmonary dysplasia
CI: Cardiac index
CO: Cardiac output
DOL: Day of life
EC: Electric cardiometry
ELBWI: Extremely low birthweight infants
GA: Gestational age
GERD: Gastroesophageal reflux disease
HSPDA: Hemodynamically significant patent ductus arteriosus
LPA: Left pulmonary artery
LUS: Lung ultrasound score
NIRS: Near-infrared spectroscopy
NSAIDs: Non-steroidal anti-inflammatory drugs
PDA: Patent ductus arteriosus
PLCS: Post ligation cardiac syndrome
PVR: Pulmonary vascular resistance
RCT: Randomized controlled trial
ROP: Retinopathy of prematurity
rSO2: Regional tissue oxygenation
SL: Surgical ligation
SV: Stroke volume
SVR: Systemic vascular resistances
TC: Transcatheter closure
VLBWI: Very low birthweight infants
